# Impact of Dupilumab on Small Airway Disease in Severe Asthma: A 12-Month Retrospective Real-World Study

**DOI:** 10.3390/arm94020014

**Published:** 2026-02-26

**Authors:** Lorenzo Carriera, Angelo Coppola, Roberto Lipsi, Stefano Baglioni, Pier-Valerio Mari, Roberto Barone, Simone Ielo, Raffaele Scala, Andrea Smargiassi, Riccardo Inchingolo, Luca Richeldi, Valeria Gambacorta, Alfredo Di Giovanni, Eugenio De Corso

**Affiliations:** 1 Facoltà di Medicina e Chirurgia, Università Cattolica del Sacro Cuore, 00168 Rome, Italy; roberto.barone01@icatt.it; 2 Department of Pulmonology and Sub-Intensive Respiratory Unit, Ospedale Santa Maria della Misericordia, 06156 Perugia, Italy; roberto.lipsi@ospedale.perugia.it (R.L.); stefano.baglioni@ospedale.perugia.it (S.B.); 3 UOC Pneumologia, Ospedale San Filippo Neri-ASL Roma 1, 00135 Rome, Italy; angelo.coppola@aslroma1.it; 4 UniCamillus, Saint Camillus International University of Health Sciences, 00131 Rome, Italy; 5 Ospedale San Carlo di Nancy, 00165 Rome, Italy; piervalerio.mari@gmail.com; 6 UOC Pneumologia e UTIP, Ospedale San Donato, USL Toscana SudEst, 52100 Arezzo, Italy; simone.ielo@uslsudest.toscana.it (S.I.); raffaele.scala@uslsudest.toscana.it (R.S.); 7 UOC Pneumologia, Dipartimento Neuroscienze, Organi di Senso e Torace, Fondazione Policlinico Universitario A. Gemelli IRCCS, 00168 Rome, Italy; andrea.smargiassi@policlinicogemelli.it (A.S.); riccardo.inchingolo@policlinicogemelli.it (R.I.); luca.richeldi@policlinicogemelli.it (L.R.); 8 Department of Medicine and Surgery, Section of Otorhinolaryngology, University of Perugia, 06123 Perugia, Italy; valeria.gambacorta@unipg.it (V.G.); alfredo.digiovanni@ospedale.perugia.it (A.D.G.); eugenio.decorso@unipg.it (E.D.C.)

**Keywords:** asthma, small airways, small-airway disease, impulse oscillometry, dupilumab

## Abstract

**Highlights:**

**What are the main findings?**

- Dupilumab significantly and sustainably improved small-airway disease (SAD) over 12 months, as demonstrated by reductions in R5–R20 and increases in FEF25–75% predicted in a real-world severe asthma cohort.
- Improvements in small-airway function were associated with better clinical control and reduced FeNO.

**What are the implications of the main findings?**

- SAD is a relevant and modifiable treatment target in severe asthma, and dupilumab may effectively address this under-recognized component of airflow limitation.
- Assessment of small-airway function may improve phenotyping, and impulse oscillometry (IOS) may represent a valuable tool for monitoring biologic treatment response.

**Abstract:**

Small-airway disease (SAD) is a key feature of severe asthma and is associated with poor symptom control and frequent exacerbations. Dupilumab has demonstrated efficacy in improving lung function and reducing exacerbations, but real-world evidence on its effects in SAD remains limited. The aim of this study is to evaluate the impact of 12 months of dupilumab treatment on SAD, clinical outcomes, and type 2 inflammation. We included 21 patients. Small-airway function was assessed by impulse oscillometry (R5–R20) and spirometry FEF25–75% predicted at baseline (T0) and after 3 (T3), 6 (T6), and 12 (T12) months of treatment. Additional assessments included FEV_1_, the Asthma Control Test (ACT), exacerbation frequency, oral corticosteroid (OCS) use, the blood eosinophil count (BEC), and fractional exhaled nitric oxide (FeNO). At baseline, 62% of patients exhibited SAD (R5–R20 > 0.07 kPa/L/s). Dupilumab treatment led to a significant and sustained improvement in small-airway function: mean R5–R20 decreased from 0.18 ± 0.17 kPa/L/s to 0.09 ± 0.07 at T12 (*p* = 0.04), while predicted FEF25–75% increased from 29.5 ± 20.8% to 47.0 ± 21.1% (*p* < 0.001). ACT scores improved from 13.1 ± 4.9 to 19.6 ± 3.8 (*p* < 0.001). FeNO levels declined from 64.1 ± 50.7 ppb to 24.8 ± 20.9 ppb (*p* = 0.01). Improvements in R5–R20 correlated with better ACT and FeNO reductions. In this real-world cohort, dupilumab significantly improved SAD, lung function, and asthma control, while reducing exacerbations, OCS dependence, and type 2 inflammation over 12 months.

## 1. Introduction

Asthma is a chronic inflammatory disorder of the airways that affects an estimated 300 million people worldwide. Among these, approximately 5–10% suffer from severe asthma, a condition defined as asthma that requires high-dose inhaled corticosteroids (ICSs) combined with a second controller (and/or systemic corticosteroids) to prevent loss of control, or that remains uncontrolled despite such therapy [1].

Small airways, defined as bronchi with an internal diameter of 2 mm or less, play a pivotal role in the pathophysiology of obstructive lung diseases [2]. In asthma, these distal airways are not passive conduits but important sites of inflammation, epithelial injury, and structural remodeling, changes that contribute to airflow limitation and ventilation heterogeneity [3]. Increasing evidence shows that small-airway dysfunction (SAD) is present across the spectrum of asthma severity and is particularly pronounced in patients with uncontrolled or severe disease, where it correlates with symptoms and exacerbation risk [4]. Multiple techniques have been employed to assess SAD. Traditional spirometric indices, such as the forced expiratory flow between 25% and 75% of forced vital capacity (FEF25–75%), provide indirect information on distal airway function, although they lack sensitivity and specificity [5]. More recently, effort-independent methods such as impulse oscillometry (IOS), particularly the measurement of resistance heterogeneity between 5 and 20 Hz (R5–R20), have become increasingly used. These techniques are more sensitive to peripheral resistance and ventilation heterogeneity and have helped reveal the extent of distal airway pathology [6]. According to the literature findings, R5–R20 baseline values > 0.07 kPa/L/s support the evidence of SAD [7].

Dupilumab, a fully human monoclonal antibody targeting the IL-4 receptor α and thereby inhibiting both IL-4 and IL-13 signaling, has emerged as an effective treatment for severe type 2 asthma [8]. Beyond its established efficacy in improving lung function and reducing exacerbations, increasing evidence indicates that dupilumab may also improve SAD. A recent post hoc analysis [9] of the phase 4 VESTIGE trial [10] demonstrated that, over a 24-week treatment period, dupilumab significantly improved small-airway function in patients with moderate-to-severe asthma compared with the placebo, as evidenced by increases in pre- and post-bronchodilator FEF25–75%, reductions in R5–R20, improvements in reactance area (AX), and decreases in airway wall thickness and air trapping. While VESTIGE provides robust randomized trial evidence, real-world studies remain essential to confirm the extent and durability of dupilumab’s benefits on SAD, and to clarify its relationship with symptom control, exacerbation reduction, and biomarker modulation.

## 2. Materials and Methods

### 2.1. Study Design and Population

This 12-month, retrospective, observational cohort study was conducted at the Asthma Center of the A. Gemelli Hospital Foundation—IRCCS, Catholic University of Sacred Heart and at the department of Pulmonology of Santa Maria della Misericordia Hospital, Perugia. All biologic-naïve patients initiating dupilumab at our severe asthma clinics between 2024 and 2025 underwent standardized clinical, functional, and laboratory assessments as part of routine care. Eligibility for step-up therapy was defined as uncontrolled disease despite optimized inhalation therapy, with blood eosinophils >150/µL and/or FeNO >25 ppb, and either ≥2 exacerbations in the previous year or oral corticosteroid dependence for ≥6 months. Patients were retrospectively included in the present analysis if they provided informed consent and completed the scheduled 12-month follow-up evaluations. A per-protocol approach was applied so that only patients with complete data were analyzed. Therefore, patients who did not provide informed consent or who did not complete follow-up were excluded from the analysis. All the included subjects received dupilumab 300 mg subcutaneously every 2 weeks after an initial loading dose of 600 mg. Demographic data were collected at baseline. Small-airway function, assessed by IOS, and respiratory function parameters measured by spirometry (including FEF25–75% predicted and RV/TLC%) were evaluated at baseline and after 3 (T3), 6 (T6), and 12 (T12) months of dupilumab treatment. Additional assessments included the Asthma Control Test (ACT), the frequency of asthma exacerbations (defined as episodes requiring systemic corticosteroid treatment), chronic oral corticosteroid (OCS) use, the blood eosinophil count (BEC), and fractional exhaled nitric oxide (FeNO). Because visits were scheduled as part of routine care, a ±2-week window was applied around each target time point to account for expected scheduling variability. This retrospective cohort study was exploratory and hypothesis-generating in nature. Considering the small sample size, the study was not powered to provide definitive efficacy conclusions but aimed to offer real-world evidence to inform future larger investigations. This study was approved by the local ethics committee and conducted in accordance with the Declaration of Helsinki. Informed consent about privacy and utilization of clinical data was obtained from all patients at baseline. Clinical data were anonymously analyzed.

### 2.2. Outcome Measurements

Pulmonary function tests were performed using the Q-Box system (COSMED—The Metabolic Company, Rome, Italy). Spirometric indices were obtained in accordance with ATS/ERS recommendations [11]. At least three acceptable maneuvers were performed, and reproducibility criteria were applied according to guideline standards [11]. FEV1 and FEF25–75% values were derived from the best acceptable FVC maneuver. Pre-bronchodilator values were used for analysis. Predicted values were calculated using Global Lung Function Initiative (GLI) reference equations [12]. Impulse oscillometry (IOS) was performed using the MasterScreen IOS system (Vyaire Medical, Mettawa, IL, USA), in accordance with manufacturer recommendations and European Respiratory Society technical standards [13]. Oscillatory measurements were obtained during tidal breathing with patients seated, wearing a nose clip, with their lips tightly sealed around the mouthpiece and cheeks firmly supported to minimize upper airway shunting. Measurements were recorded over 30 s trials. At least three technically acceptable and reproducible recordings were obtained. Mean values from acceptable recordings were used for analysis. The frequency of the waves delivered in IOS ranges from 5 to 30 Hz. Resistance at 5 Hz (R5) and 20 Hz (R20) was measured. Small-airway dysfunction (SAD) was defined as R5–R20 > 0.07 kPa/L/s, in line with published evidence [7,14,15], and this cut-off was applied consistently at all study visits. FeNO was measured using a chemiluminescence analyzer (NIOX Flex, Aerocrine AB, Solna, Sweden) at an exhalation flow of 50 mL/s.

### 2.3. Statistical Analysis

Statistical analyses were performed and figures were generated using Prism 11 (Graphpad Software Inc., La Jolla, CA, USA). Continuous variables are presented as mean ± standard deviation (SD) when normally distributed or as median and interquartile range (IQR) when non-normally distributed. Categorical variables are expressed as absolute numbers and percentages (n, %). Normality of continuous variables was assessed using both visual methods (histograms and Q-Q plots) and the Shapiro–Wilk test (*p* > 0.05 indicating approximate normal distribution). Given the repeated assessments at baseline (T0), 3 months (T3), 6 months (T6), and 12 months (T12), longitudinal changes over time were analyzed using repeated-measures analysis of variance (ANOVA) for normally distributed variables. When the assumption of sphericity was violated, Greenhouse–Geisser correction was applied. Bonferroni’s multiple comparisons test was adopted to compare means for continuous variables. Non-normally distributed longitudinal variables were analyzed using the Friedman test with Holm-adjusted post hoc comparisons. Categorical repeated measures were compared using McNemar’s test. Correlations between continuous variables were assessed using Pearson’s correlation coefficient for approximately normally distributed data and Spearman’s rank correlation coefficient for non-normally distributed variables. A *p* value lower than 0.05 was considered to be significant.

## 3. Results

A total of 21 patients were enrolled, of whom 13 were recruited at A. Gemelli Hospital Foundation—IRCCS, Catholic University of Sacred Heart and 8 patients at the department of Pulmonology of Santa Maria della Misericordia Hospital, Perugia. The mean (±SD) age was 56.3 ± 13.6 years, with a mean BMI of 24.8 ± 2.48 kg/m^2^. Ten subjects (47.6%) were female, and nine (42.8%) were either current or former smokers. All participants were receiving maintenance inhaled therapy with medium-to-high-dose ICS/LABA; additionally, 16 patients (76%) were treated with a LAMA. Comorbid nasal polyposis was present in 11 patients (52.3%). Other comorbidities included bronchiectasis, which was present in 5 patients (23.8%), allergic sensitization in 12 patients (57.1%), atopic dermatitis in 2 patients (9.5%), and gastroesophageal reflux disease (GERD) in 9 patients (42.9%). All patients were biologic-naïve at dupilumab initiation. At baseline, small-airway dysfunction (SAD), defined as R5–R20 > 0.07 kPa/L/s, was detected in 13 subjects (62%). The median (IQR) R5–R20 value for the overall cohort was 0.17 (0.04–0.26) kPa/L/s. Baseline spirometry showed a mean FEV_1_ of 1.91 ± 0.72 L, corresponding to 61.6 ± 20.9% of pred., with a mean Tiffeneau index of 56.0 ± 14.4%. Other functional parameters included a mean FEF25–75% pred. of 29.5 ± 20.8%, TLC% pred. of 99.2 ± 16.2%, and RV/TLC% of 49.0 ± 22.9%. Regarding disease control, the mean ACT score was 13.1 ± 4.9 points. Eight patients (38%) reported at least two OCSs requiring exacerbation during the previous year, while four patients (19%) were on maintenance OCS. The median (IQR) blood eosinophil count was 420 (175–525) cells/µL, and the mean (±SD) FeNO was 64.1 ± 50.7 ppb. Study population characteristics at baseline are presented in Table 1, while respiratory function parameters at baseline are presented in Table 2.

**Figure 1:**
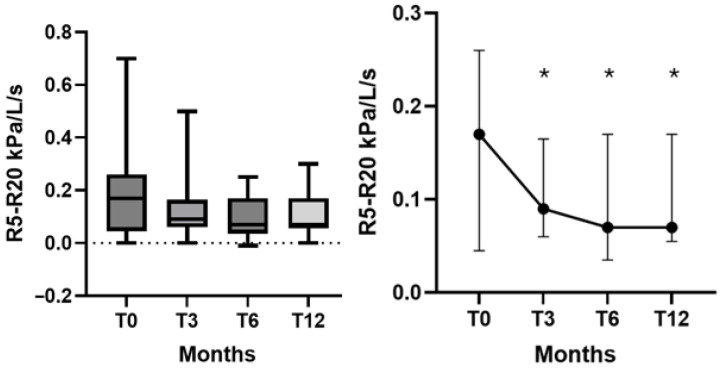
Dupilumab effectiveness on R5–R20 values at T0, T3, T6 and T12. Left panel: Box plots showing median, IQR and maximum/minimum values for R5–R20 (kPa/L/s). Right panel: Trends of the median and IQR of R5–R20 from baseline T0 to T12. * = *p* < 0.05.

A progressive reduction in R5–R20 values was observed over the 12-month treatment period. The median (IQR) R5–R20 decreased from 0.17 (0.04–0.26) kPa/L/s at baseline to 0.09 (0.06–0.16) at T3 (*p* = 0.04), 0.07 (0.03–0.17) at T6 (*p* = 0.04), and 0.07 (0.05–0.17) at T12 (*p* = 0.04) (Figure 1).

**Figure 2:**
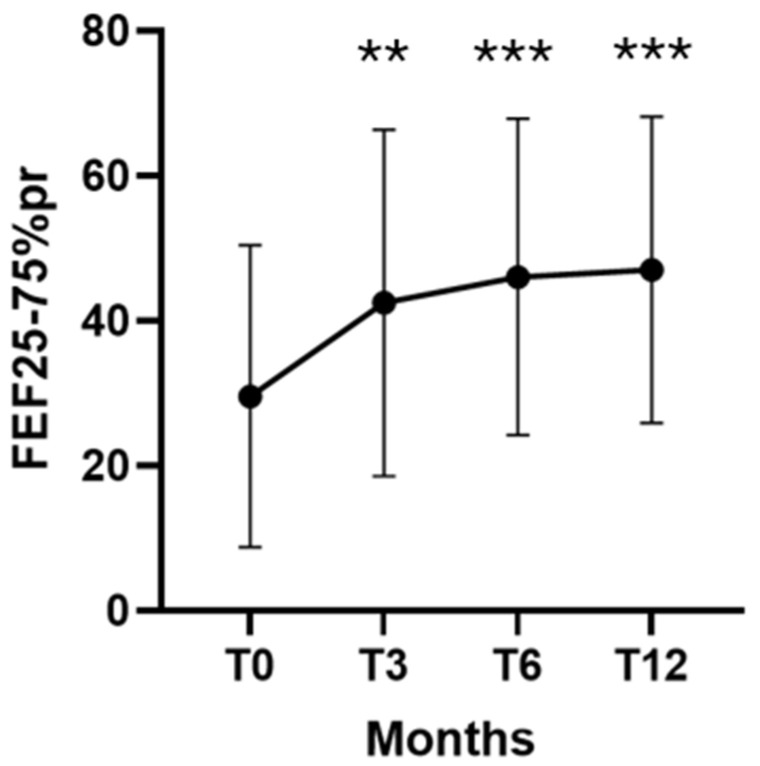
Dupilumab effectiveness on FEF25–75% predicted at T0, T3, T6 and T12. Temporal trends of FEF25–75%pr values. Data are presented as mean (±SD). ** = *p* < 0.01; *** = *p* < 0.001.

The number of patients with SAD decreased from 13 (62%) at baseline to 11 at T3, 8 at T6, and 7 (33%) at T12; this reduction showed a trend toward significance (*p* = 0.058). Improvement in small-airway function was further confirmed by changes in predicted FEF25–75%, which increased from a baseline mean (±SD) of 29.5 ± 20.8% to 42.4 ± 23.8% at T3 (*p* = 0.001), 46.0 ± 21.8% at T6 (*p* = 0.0002), and 47.0 ± 21.1% at T12 (*p* = 0.0003) (Figure 2).

**Figure 3:**
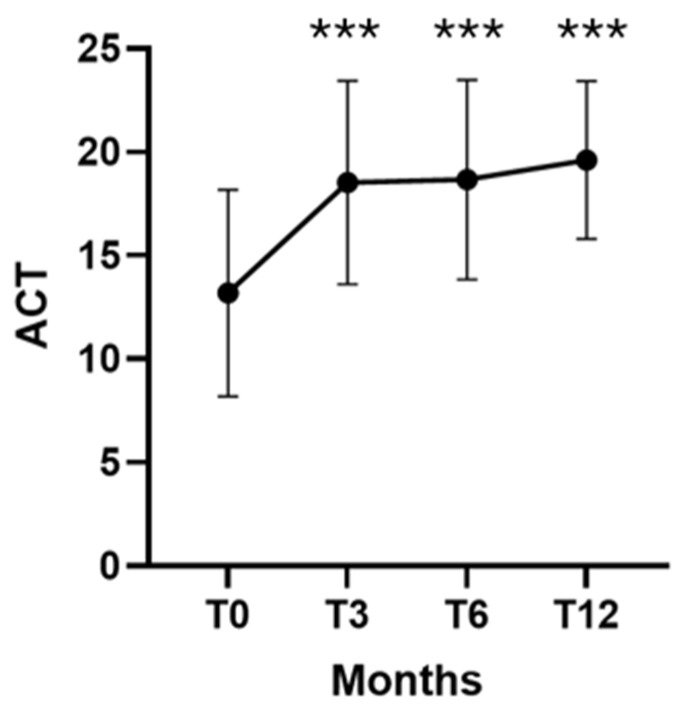
Dupilumab effectiveness on ACT values at T0, T3, T6 and T12. Temporal trends of ACT values. Data are presented as mean (± SD). *** = *p* < 0.001.

FEV_1_% pred. significantly improved from a baseline of 61.6 ± 20.9% to 79.6 ± 21.0% at T3 (*p* < 0.001), 76.8 ± 24.2% at T6 (*p* = 0.004), and 81.5 ± 22.2% at T12 (*p* = 0.002). ACT scores improved markedly, increasing from 13.1 ± 4.9 at baseline to 18.5 ± 4.9 at T3 (*p* <0.001), 18.6 ± 4.8 at T6 (*p* <0.001), and 19.6 ± 3.8 at T12 (*p* < 0.001) (Figure 3).

Notably, all four patients receiving OCS discontinued treatment within the first six months of dupilumab therapy (one at T3, three at T6) and maintained OCS withdrawal through T12 (*p* < 0.001). The number of patients experiencing exacerbations fell from eight (38%) in the year prior to enrollment to one (4.7%) at T12 (*p* = 0.03). FeNO levels decreased significantly from 64.1 ± 50.7 ppb at baseline to 28.5 ± 23.6 ppb at T3 (*p* = 0.04), 29.8 ± 19.2 ppb at T6 (*p* = 0.04), and 24.8 ± 20.9 ppb at T12 (*p* = 0.03). All results are reported in Table 3.

**Figure 4:**
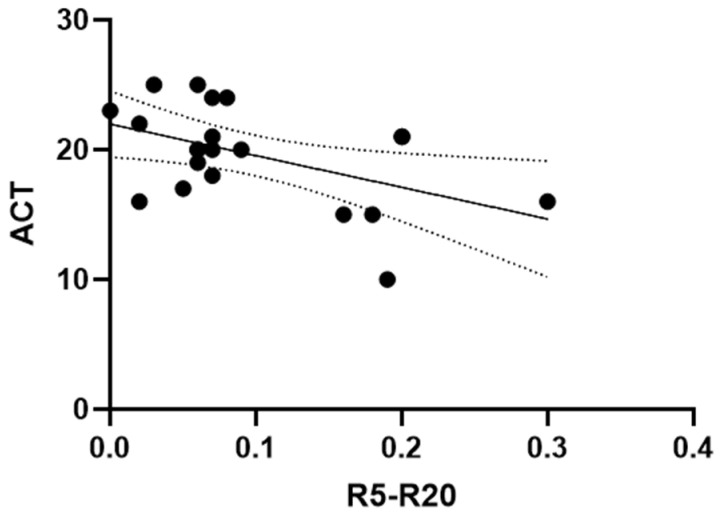
Correlation between ACT and R5–R20 after 12 months of dupilumab treatment. Scatter plot showing the association between changes in R5–R20 and changes in ACT after 12 months. The solid line represents the linear regression fit, and the dotted lines indicate the 95% confidence interval. A weak-to-moderate inverse correlation was observed (Pearson’s r = −0.49, 95% CI −0.76 to −0.07; *p* = 0.02). The regression equation was y = −24.39 x + 21.

An inverse correlation was observed between R5–R20 and ACT values at 12 months (Pearson’s r = −0.49, *p* = 0.02) (Figure 4).

**Figure 5:**
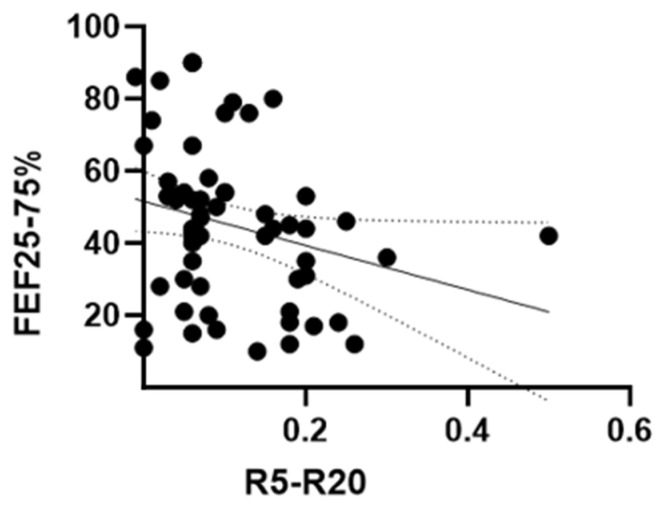
Correlation between FEF25–75%pred and R5–R20 during dupilumab treatment. Scatter plot showing the association between changes in R5–R20 and changes in FEF25–75%pred over 12 months. The solid line represents the linear regression fit, and the dotted lines indicate the 95% confidence interval. A weak correlation was observed (Pearson’s r = −0.24, 95% CI −0.46 to −0.001; *p* = 0.04). The regression equation was y = −61.34 x + 51.64.

The relationship between R5–R20 and FEF25–75% pred. was assessed longitudinally across the full 12-month observation period, and we found a weak correlation (Pearson r: −0.24, *p*: 0.04) (Figure 5).

**Figure 6:**
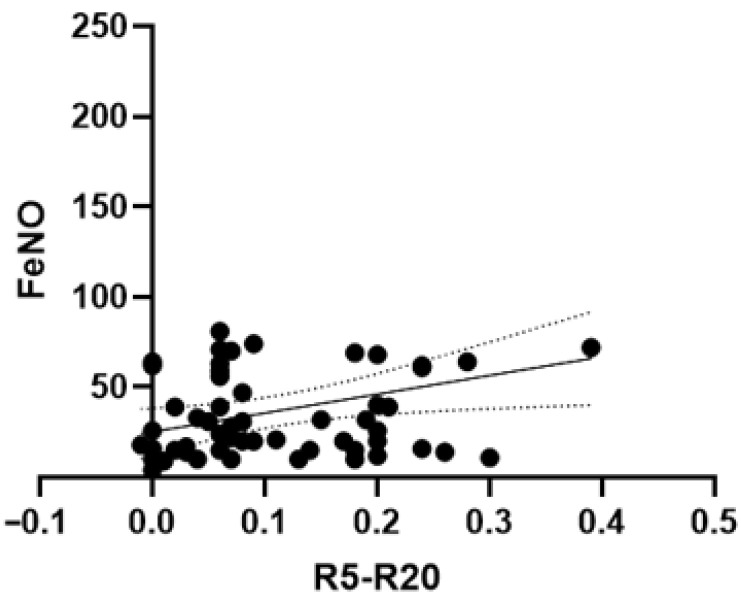
Correlation for change from baseline to T12 in FeNO levels with change from baseline to T12 in R5–R20 values. Scatter plot showing the association between changes in R5–R20 and changes in FeNO over 12 months. The solid line represents the linear regression fit, and the dotted lines indicate the 95% confidence interval. A weak correlation was observed (Pearson’s r = 0.29, 95% CI 0.04 to 0.5; *p* = 0.03). The regression equation was y = 104.1 x + 25.35.

We also observed a correlation between FeNO levels and R5-R20 changes from baseline to T12 (Pearson r: −0.27, *p* = 0.03) (Figure 6).

## 4. Discussion

In this real-world cohort of patients with severe asthma, dupilumab treatment was associated with a progressive and sustained improvement in small-airway function over 12 months, as demonstrated by reductions in IOS-derived R5–R20 values and parallel increases in FEF25–75%. At baseline, 62% of patients exhibited SAD despite optimized inhaled therapy, confirming the high prevalence of distal airway involvement in severe asthma. IOS showed a significant reduction in R5–R20 values as early as 3 months, with progressive improvement through 12 months, while the proportion of patients with SAD fell to 33% by study end. These improvements in SAD were accompanied by significant modifications in lung function (FEV_1_), better symptom control (ACT), reduced type 2 airway inflammation (FeNO), and clinical outcomes, including the exacerbation rate and OCS-sparing effect. The correlation between R5–R20 and ACT at 12 months suggests that distal airway improvements translated directly into better symptom control. This relationship supports the clinical relevance of SAD, previously regarded as a “silent zone,” and underscores its contribution to patients’ daily symptom burden. The mean ACT score improved by at least 3 points, which is the minimal clinically important difference (MCID) [16]. FeNO levels, elevated at baseline, declined significantly during treatment and correlated with R5–R20 reductions, suggesting that suppression of type 2 inflammation contributes to improved small-airway function. The elevation in BEC observed in our cohort is in line with previous evidence showing that dupilumab may induce transient or persistent peripheral eosinophilia, likely due to inhibition of IL-4/IL-13 signaling and reduced eosinophil migration into tissues [17,18]. Therefore, BEC may not reliably reflect airway type 2 inflammation during dupilumab therapy and should be interpreted cautiously. Rather than serving as a marker of treatment efficacy, eosinophil counts should primarily be monitored to detect clinically significant elevations [19]. Dupilumab blockade of IL-4 and IL-13, cytokines that promote distal airway inflammation, mucus hypersecretion, and structural changes may be the key to the observed improvements in small-airway function [20]. In particular, the dissolution of mucus plugs resulting from the blockade of IL-13 significantly improved airway caliber and ventilation [21]. The effect of dupilumab on SAD was previously shown by other small retrospective or prospective studies. Its efficacy has also been demonstrated in reversing SAD in comparison with other biologics. A post hoc analysis from two prospective phase IV clinical trials investigating the effect of benralizumab and dupilumab in patients with type 2 high poorly controlled severe asthma, where oscillometry was a secondary endpoint, demonstrated a superior efficacy with dupilumab compared to benralizumab in terms of peripheral airway resistance and compliance [22]. A retrospective analysis of severe asthma patients with abnormal baseline oscillometry-defined SAD treated with dupilumab experienced improvements in oscillometry outcomes after 4.5 months of biologic therapy [23]. In our study, R5–R20 values improved in just 3 months, with progressive improvement through 12 months. A retrospective study on 20 patients by Greig et al. [24] showed improvements in the respiratory function parameters measured through spirometry and oscillometry, type 2 biomarkers and symptom control. No significant correlations were found between the ratios and the T2 biomarkers or symptom score. In a longer follow-up, we observed a weak-to-moderate correlation between R5–R20 and ACT, suggesting that distal airway improvements may result into better symptom control. In addition, reductions in type 2 inflammation biomarkers, particularly FeNO values, were associated with improvements in distal airway function. Although the observed associations between improvements in small-airway indices and clinical/inflammatory outcomes were statistically significant, their magnitude was weak to moderate and should be interpreted cautiously given the limited sample size and the exploratory nature of correlation analyses. A Japanese multi-center prospective cohort study on 99 severe asthma patients treated with dupilumab showed improvements in lung function parameters over 24 weeks. FEF25–75% significantly improved in SAD patients, but oscillometric parameters only improved in patients with large- and small-airway dysfunction [25]. Our study showed improvements in both IOS and FEF25–75% and identified an association between these measures. Traditionally, the correlation between IOS and conventional spirometry is weak. FEF25–75% is the most commonly used spirometric index to assess peripheral airway obstruction in routine clinical practice. Its role in predicting peripheral obstruction, however, is still controversial, since a correlation with direct measurement through IOS is not always observed [4]. To conclude, Stewart et al. demonstrated the effect of dupilumab over 12 weeks in a cohort of severe asthma patients, with improvements in R5–R20, AX, FEF25–75 and ACQ5. It is interesting that after 12 weeks of washout from the monoclonal antibody, there was a noticeable trend towards worsening of SAD outcomes [26]. Our findings are consistent with those of the VESTIGE trial, which demonstrated that dupilumab significantly improves small-airway function, as assessed by FEF25–75%, oscillometric indices, and imaging measures after 24 weeks of treatment. In line with VESTIGE, we observed substantial improvements in FEF25–75% and reductions in oscillometric markers of SAD, together with parallel improvements in symptom control and type 2 inflammation. Importantly, in this small, uncontrolled real-world cohort, these trends were maintained over 12 months, providing supportive evidence for the persistence of distal airway improvements during continued dupilumab treatment. In addition, while VESTIGE focused primarily on physiological and imaging endpoints, our real-world cohort provides complementary clinical evidence, showing marked reductions in exacerbations and complete withdrawal of maintenance OCS in all treated patients. Taken together, these data strengthen the evidence that dupilumab not only improves small airway physiology in the short term, as demonstrated in VESTIGE, but also translates into a sustained clinical benefit and steroid-sparing effects over longer treatment periods. Nevertheless, some limitations should be acknowledged. First, the study sample was relatively small and derived from a two-center cohort within a single country, which may limit the generalizability to broader severe asthma populations with different clinical phenotypes and healthcare settings. Second, the absence of a comparator group (e.g., patients receiving standard care or initiating another biologic) limits the attribution of the observed changes exclusively to dupilumab and precludes definitive causal inference. Third, IOS is not yet routinely available in many clinical settings and variability in acquisition/quality control procedures may affect the comparability across centers. Finally, a longer follow-up and larger comparative studies are warranted to confirm the durability of benefits beyond one year and to better define the role of small-airway assessment in treatment monitoring.

## 5. Conclusions

Our real-world findings suggest a beneficial association between dupilumab treatment and improved small-airway function in patients with severe asthma. Given the expanding role of asthma biologics [27,28], targeting small-airway dysfunction with dupilumab may represent an effective therapeutic strategy, as improvements in distal airway mechanics were closely associated with better disease control, fewer exacerbations, and reduced type 2 inflammation. While spirometry remains the cornerstone of functional assessment, IOS offers greater sensitivity in detecting peripheral airway involvement and may provide a complementary perspective in patients with severe asthma. The gradual reduction in R5–R20, in parallel with symptomatic and clinical improvements, supports its role as a potential biomarker for treatment response to biologics. Larger prospective studies with a longer follow-up are needed to confirm these observations.

## Figures and Tables

**Table 1:** Study population characteristics at baseline (n = 21).

Age (years)	56.3 (±13.6)
Gender, n (%)	Female: 10 (47.6) Male: 11 (52.4)
BMI (kg/m^2^)	24.8 (±2.5)
Smoking history, n (%)	Never: 12 (57.2) Former: 4 (19) Current: 5 (23.8)
R5–R20 > 0.07 kPa/L/s (SAD), n (%)	13 (62)
ACT score	13.1 (±4.9)
BEC (cells/µL)	420 (175–525)
FeNO (ppb)	64.1 (±50.7)
Patients ≥2 AE requiring OCS in previous year, n (%)	8 (38)
Maintenance OCS, n (%)	4 (19)
Nasal polyposis, n (%)	11 (52.3)
Atopy, n (%)	12 (57.1)
Atopic dermatitis, n (%)	2 (9.5)
Bronchiectasis, n (%)	5 (23.8)
Gastroesophageal reflux disease (GERD), n (%)	9 (42.9)

All data are presented as mean(±SD) or median (IQR) for BEC. Gender, smoking history, SAD patients with R5–R20 > 0.07 kPa/L, patients with ≥ 2 AE requiring OCS in the previous year, patients with maintenance OCS therapy and comorbidities are presented as N(%).

**Table 2:** Study population respiratory function at baseline (n = 21).

R5–R20 (kPa/L/s)	0.17 (0.04–0.26)
FEV1 (L)	1.91 (±0.72)
FEV1%pred.	61.6 (±20.9)
FEV1/FVC %	56.0 (±14.4)
FEF25–75%pred.	29.5 (±20.8)
TLC %pred.	99.2 (±16.2)
RV/TLC %	48.0 (±21.9)

Data are presented as mean (±SD) and median (IQR) for R5–R20 values.

**Table 3:** Study results.

	T0	T3	*p*-Value T0–T3	T6	*p*-Value T0–T6	T12	*p*-Value T0–T12
R5–R20 (kPa/L/s)	0.17 (0.04–0.26)	0.09 (0.06–0.16)	**0.04 _a_**	0.07 (0.03–0.17)	**0.04 _a_**	0.07 (0.05–0.17)	**0.04 _a_**
FEF25–75%pred.	29.5 (±20.8)	42.4 (±23.8)	**0.001 _b_**	46.0 (±21.8)	**<0.001 _b_**	47.0 (±21.1)	**<0.001 _b_**
RV/TLC%	48.0 (±21.9)	42.4 (±15.5)	0.1 **_b_**	40.0 (±8.8)	0.3 **_b_**	35.4 (±7.0)	**0.04 _b_**
FEV1%pred.	61.6 (±20.9)	79.6 (±21.0)	**<0.001 _b_**	76.8 (±24.2)	**0.004 _b_**	81.5 (±22.2)	**0.002 _b_**
ACT	13.1 (±4.9)	18.5 (±4.9)	**<0.001 _b_**	18.6 (±4.8)	**<0.001 _b_**	19.6 (±3.8)	**<0.001 _b_**
BEC (cell/mm^3^)	420 (175–525)	830 (185–1005)	**0.04 _a_**	800 (340–1630)	**0.04 _a_**	780 (200–910)	0.3 **_a_**
FeNO (ppb)	64.1 (±50.7)	28.5 (±23.6)	**0.04 _b_**	29.8 (±19.2)	**0.04 _b_**	24.8 (±20.9)	**0.03 _b_**

All data are presented as mean (± SD) or median (IQR) for R5–R20 and BEC. _a_: Friedman test with post hoc pairwise comparisons. _b_: Repeated-measures ANOVA.

## Data Availability

All data generated or analyzed during this study are included in this article. Further enquiries can be directed to the corresponding author.

## Abbreviations

The following abbreviations are used in this manuscript:

ACT	Asthma Control Test
BEC	Blood Eosinophil Count
BMI	Body Mass Index
FEF25–75%	Forced Expiratory Flow between 25% and 75% of Vital Capacity;
FeNO	Fractional Exhaled Nitric Oxide
FEV1/FVC	Forced Expiratory Volume in 1 s/Forced Vital Capacity
FEV1	Forced Expiratory Volume in 1 s % of Predicted
FVC	Forced Vital Capacity
ICS	Inhaled Corticosteroid
ICS/LABA	Inhaled Corticosteroids/Long-Acting Beta Agonists
IOS	Impulse Oscillometry
IQR	Interquartile Range
LAMA	Long-acting Muscarinic Agonist
OCS	Oral Corticosteroid
R5–R20	Resistance at 5 Hz and 20 Hz
RV/TLC	Residual Volume/Total Lung Capacity
SAD	Small-Airway Disease
SD	Standard Deviation
TLC	Total Lung Capacity
